# A Soluble Acetylcholinesterase Provides Chemical Defense against Xenobiotics in the Pinewood Nematode

**DOI:** 10.1371/journal.pone.0019063

**Published:** 2011-04-27

**Authors:** Jae Soon Kang, Dae-Weon Lee, Young Ho Koh, Si Hyeock Lee

**Affiliations:** 1 Research Institute for Agriculture and Life Science, Seoul National University, Seoul, Korea; 2 Ilsong Institute of Life Science, Hallym University, Anyang, Korea; 3 Department of Agricultural Biotechnology, Seoul National University, Seoul, Korea; University of Cambridge, United Kingdom

## Abstract

The pinewood nematode genome encodes at least three distinct acetylcholinesterases (AChEs). To understand physiological roles of the three pinewood nematode AChEs (BxACE-1, BxACE-2, and BxACE-3), BxACE-3 in particular, their tissue distribution and inhibition profiles were investigated. Immunohistochemistry revealed that BxACE-1 and BxACE-2 were distributed in neuronal tissues. In contrast, BxACE-3 was detected from some specific tissues and extracted without the aid of detergent, suggesting its soluble nature unlike BxACE-1 and BxACE-2. When present together, BxAChE3 significantly reduced the inhibition of BxACE-1 and BxACE-2 by cholinesterase inhibitors. Knockdown of BxACE-3 by RNA interference significantly increased the toxicity of three nematicidal compounds, supporting the protective role of BxACE-3 against chemicals. In summary, BxACE-3 appears to have a non-neuronal function of chemical defense whereas both BxACE-1 and BxACE-2 have classical neuronal function of synaptic transmission.

## Introduction

Acetylcholinesterase (AChE, EC 3.1.1.7) plays a critical role in terminating nerve impulses by hydrolyzing the neurotransmitter, acetylcholine (ACh) in the cholinergic nervous system of most animals [1]. AChE is also reported to be distributed in a variety of non-neuronal tissues in vertebrates [2], [3], [4]. Different from vertebrates having two cholinesterases, AChE and butyrylcholinesterase (BuChE, EC 3.1.1.8) [5], [6], most invertebrates, such as arthropods and nematodes, have only AChEs [7], [8].

*Caenorhabditis elegans*, a free-living nematode, has four *ace* genes encoding different AChE types (ACE-1, ACE-2, ACE-3 and ACE-4). Each AChE showed different pharmacological properties [7] and localization pattern in tissue and cells [9], [10], suggesting their multiple physiological functions. Studies using null mutant worms revealed that both ACE-1 and ACE-2 are major functional enzymes with mutually compensating functions [11], [12] whereas ACE-3 does not compensate for the role of ACE-1 or ACE-2. Moreover, kinetics or inhibition assays suggested that ACE-3 is associated with non-classical synaptic functions [13], [14]. Biochemical properties of ACE-3 were also reported in several plant parasitic nematodes, including *Heterodera glycines*, *Meloidogyne arenaria* and *M. incognita* [15], [16].

In our previous study, three AChEs (BxACE-1, BxACE-2 and BxACE-3) were identified from the pinewood nematode, *Bursaphelenchus xylophilus*, a serious phytopathogen to pine trees in several countries [17] and their molecular and kinetic properties were characterized using recombinant enzymes expressed *in vitro*. BxACE-1 and BxACE-2 showed similar biochemical properties, including the catalytic efficiency and the substrate and inhibitor specificities, to those of other nematodes. Unlike BxACE-1 and BxACE-2, which appear to play common but non-overlapping synaptic functions, BxACE-3 exhibited several unique features. First, no GPI-anchoring sequence or the H-type hydrophobic sequence was predicted from cDNA, suggesting the soluble nature BxACE-3. Second, BxACE-3 showed the highest transcription level but lowest catalytic efficiency, indicating that it may not function in postsynaptic transmission. Third, the sensitivity to various anti-cholinesterase inhibitors was much lower in BxACE-3, showing unusual inhibition properties compared to typical neuronal AChEs. Based on these findings, it was hypothesized that BxACE-3 is not likely involved in postsynaptic transmission but rather has non-neuronal functions [17].

In this study, to further understand the physiological roles of BxACEs, BxACE-3 in particular, we investigated their tissue distribution patterns via immuohistochemistry, *in vitro* inhibition profiles in the presence or absence of BxACE-3 and the organophosphate inhibition sensitivity of the nematodes when expression of BxACE-3 was knocked down by RNA interference (RNAi). We provided some lines of evidence that BxACE-3 has a role as bioscavenger against anti-AChEs, thereby providing non-neuronal functions of chemical defense. In addition, we demonstrated that BxACE-3 interacts with pine resin terpenes and postulated that *B. xylophilus* has evolved the chemical defense system via BxACE-3 against the endogenous anti-AChE terpene compounds. 

## Materials and Methods

### Nematodes

*B. xylophilus* was collected from the Jinju in Korea by the method described in previous study [17] and identified by real-time species-specific PCR [18]. Identified nematode was reared on a lawn of *Botrytis cinerea* cultured on PDA plates (media-grown propagative mixed stage, MGPS) at 28°C for up to a week. Fresh nematode washed by M9 buffer [19] was used immediately after separation from plates.

### In vitro expression of BxACEs and generation of anti-BxACE polyclonal antibodies (BxACEPab)

Recombinant BxACEs were expressed by baculovirus system described in previous studies and their activity was verified by kinetics [17]. Immunogens for polyclonal antibody production were expressed using a bacterial expression system. cDNA fragments encoding 100 amino acids from the N-terminus of each AChE but excluding the signal peptide sequence were inserted into the pET28a(+) expression vector (Merck, Darmstadt, Germany) and then cloned into *Escherichia coli* BL21(DE3). Immunogens were expressed by IPTG induction, and then purified using a His-tag column. The purified antigens were injected into a rabbit three times, and BxACEPabs were obtained (Ab Frontier, Seoul, Korea). BxACEPabs were purified by an affinity chromatography column using the respective antigens.

### Immunohistochemistry

MGPS of *B. xylophilus* was used for immunohistochemistry of BxACEs. A whole-body immunohistochemistry procedure was conducted using the tube-fixation protocol according to Wormbook [20]. The nematodes were rinsed with M9 buffer more than three times and fixed with 4% paraformaldehyde after freeze-fracturing with liquid nitrogen. Subsequently, β-mercaptoethanol and collagenase (type VII, Sigma-Aldrich, St. Louis, MO) were added to increase the permeability of the antibody. The collagenase-treated nematodes were blocked in 10% goat serum albumin (Jackson ImmunoResearch, West Grove, PA) in antibody buffer (pH 7.2). The BxACEPabs and anti-rabbit Alexa568 (Molecular probes, Eugene, OR) were added successively. A mixture of BxACEPab and the target recombinant BxACE (1 5 w/w) was used as a negative control, whereas mixtures with BxACEPab and the other recombinant BxACEs (1 5 w/w) were used for a positive control. The mixtures were pre-absorbed for 6 hr at room temperature. The nematodes treated with primary and secondary antibodies were mixed with Vectashield (Vector, Burlingame, CA) and mounted on glass slides. The whole-mount samples were photographed on a Zeiss LSM510 (Carl Zeiss, Oberkochen, Germany) and IX71 inverted optical microscope (Olympus, Tokyo, Japan). Digital images were processed using an LSM image browser (Carl Zeiss) and Adobe Photoshop (Adobe Inc, San Jose, CA). The nervous system anatomy of *B. xylophilus* was based on other model nematodes, including *C. elegans* and *Ascaris suum*, because there is a remarkable conservation of neuronal morphology in nematodes despite large differences in size and habitat [21].

### Electrophoresis and Western blotting

To determine whether BxACEs were associated with the membrane, proteins from *B. xylophilus* were extracted using an ultrasonicator, Sonifier 450 (Branson Ultrasonics, Danbury, CT) in 0.1 M Tris-HCl (pH 7.8) containing 20 mM NaCl in the presence or absence of 0.5% Triton-X. Native PAGE was conducted at 120 V for 90 min at 4°C, and the gel was electro-blotted to a nitrocellulose membrane (GE Healthcare, Uppsala, Sweden). Each BxACE was detected using BxACEPabs.

### In vitro inhibition assay

Acetylthiocholine iodide (ASChI), 5,5′-dithiobis(2-nitro-benzonic acid) (DTNB), bovine serum albumin (BSA), α-pinene and limonene were purchased from Sigma-Aldrich. Insecticides, including dichlorvos, chlorpyrifos-oxon, and carbofuran, were purchased from ChemService (West Chester, PA).

To investigate the effects of BxACE-3 on the inhibition of BxACE-1 and BxACE-2, BxACE-1 and BxACE-2 were mixed in a ratio of 6:1, based on their transcription levels in the pine-grown propagative stage (PGPS) [17] to simulate the *in vivo* state of BxACEs, and used in an inhibition assay. Five concentrations of dichlorvos, chlorpyrifos-oxon and carbofuran were directly added to the mixture of BxACE-1 and BxACE-2. Otherwise, the inhibitors were first pre-incubated with BxACE-3 for 10 min and then added to the mixture of BxACE-1 and BxACE-2. In the final BxACE mixture, the molar ratio of BxACE-1:BxACE-2:BxACE-3 was 6:1:18, on the basis of their respective transcript levels in the PGPS [17]. A mixture containing all three BxACEs but no inhibitors was used as control. In another control, the same molar amount of BSA instead of BxACE-3 was added to the mixture of BxACE-1 and BxACE-2 to measure the nonspecific interaction of BSA with inhibitors. The final concentrations of ASChI and DTNB were 1 mM and 0.4 mM, respectively. The remaining activity was measured after 5-min incubation with inhibitor as described above, and IC_50_ was calculated using the Polo Plus program (Ver. 2.0, Leora software, Petaluma, CA). An equality analysis between different treatments was conducted using GLM repeated measures ANOVA in the SPSS program (Version 12.0 K for Windows, SPSS Inc., Chicago, IL).

To determine the anti-AChE activity of α-pinene and limonene, each BxACE sample was incubated with six serially diluted concentrations of the test chemical in 0.1 M Tris-HCl buffer (20 mM NaCl and 1% acetone, pH 7.8) containing ASChI (final 1 mM) and DTNB (final 0.4 mM). The remaining AChE activity was measured as described above.

### Bxace-3 RNA interference (RNAi) and nematode bioassay

For the amplification of DNA templates used for dsRNA synthesis, primers containing T7 polymerase promoter sequence (Table S1) were designed using the BLOCK-iT^TM^ RNAi designer (https://rnaidesigner.invitrogen.com/rnaiexpress, Invitrogen, Carlsbad, CA). The pQE30 plasmid sequence, which does not exist in *B. xylophilus*, was used as the negative control. A PCR product was amplified using *Ex Taq* polymerase (Takara, Shiga, Japan), from which double-strand RNA (dsRNA) was prepared using MEGAscript RNAi Kit (Ambion, Austin, Texas). Approximately 300–500 mixed-stage nematodes were soaked in 20 µl RNAi solution (0.25x Mg^2+^-free M9 buffer) containing 3 mM spermidine (Sigma-Aldrich), 0.05% gelatin (Sigma-Aldrich), lipofectin (Invitrogen) and dsRNA (4 µg/μl).

Chlorpyrifos-methyl and fenamiphos were purchased from ChemService. After incubation in dsRNA solution for 1 day at 30°C, approximately 50–100 nematodes were treated with a diagnostic dose of chlorpyrifos-methyl, dichlorvos and fenamiphos (LC_50_ value, unpublished) by soaking at RT and mortality was determined at 24 hr post-treatment. Total RNA was extracted from remaining nematodes using TRI reagent (MRC, Cincinnati, OH) and used for qPCR to determine the extent of target gene knockdown. The qPCR was conducted by the procedure described in previous studies [17]. Bioassay and qPCR were conducted in three biological repeats.

## Results and Discussion

### BxACE-3 is soluble and distributed in some specific tissues

**Figure 1:**
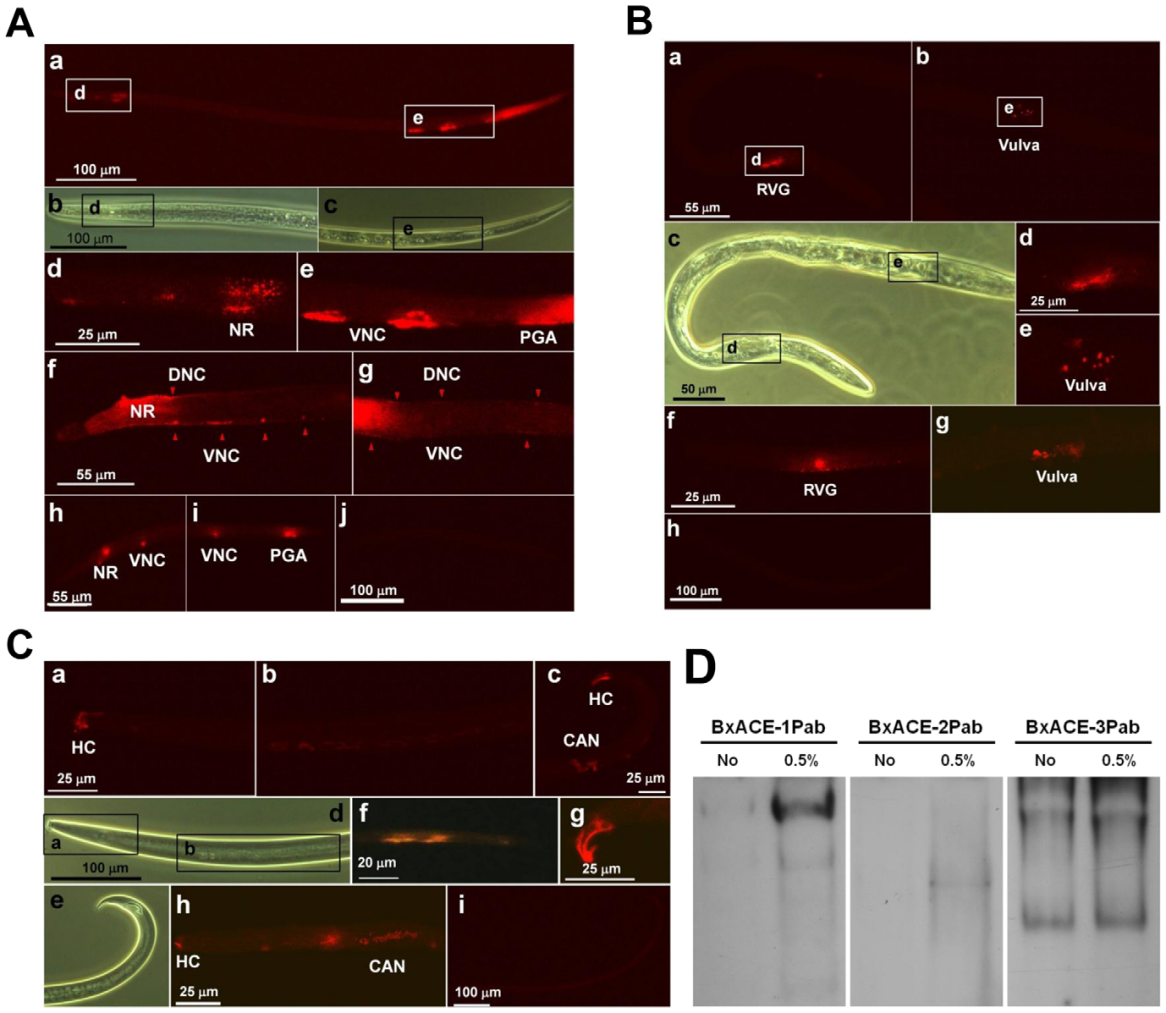
The localization pattern (A, B and C) and solubilization feature (D) of the three BxAChEs. (**A**) BxACE-1. Nematodes from (a) to (e) were female adults, and those from (f) to (j) were larvae. a, BxACE-1 is shown around the pharynx (left white box), the putative ventral nerve cord (VNC, right white box) and the tail. b and c, The head and tail regions photographed using an optical microscope. The black boxes indicate the regions in which BxACE-1 was observed. d and e, The head and tail regions of the nematode. BxACE-1 was observed at the putative nerve ring (NR) located around the pharynx (d), VNC and the putative preanal ganglia (PGA) regions (e). f and g, The head and middle body regions of the nematode. BxACE-1 was observed at the putative NR, VNC and dorsal neuron cord (DNC). The red triangles indicate BxACE-1 in the VNC and DNC. h and i, Positive control. BxACE-1 was observed at the putative NR, VNC and PGA regions. (h) and (i) are the head and tail regions, respectively. j, Negative control, in which no signal was detected. (**B**) BxACE-2. The nematodes were female adults. a and b, BxACE-2 was observed around the post-NR (a) and putative vulva (b). c, Nematodes of (a) and (b) photographed using an optical microscope. The black boxes indicate the regions in which BxACE-2 was detected. d and e, Detailed photographs of white boxes in (a) and (b). f and g, Positive control. BxACE-2 was observed around the post-NR (f) and putative vulva (g). h, Negative control, in which no signal was detected. (**C**) BxACE-3. The nematodes were male adults (a–e and g–i) and larvae (f). a, BxACE-3 was observed at putative hypodermal cells (HC) located at the end of head. b, BxACE-3 was observed at the putative intestinal regions. c, BxACE-3 was observed at the putative canal-associated neuron (CAN), the DNC and HCs at the tip of the tail. d, Nematodes of (a) and (b) photographed using an optical microscope. The black boxes indicate the regions in which BxACE-3 was detected. e, Nematode of (c) photographed using an optical microscope. f, g and h, Positive controls. BxACE-3 was observed at the putative intestinal region (f), (h), HCs of tail (g), and HCs of head and intestinal regions (h). i, Negative control, in which no signal was detected. (**D**) Detection of each BxACE using corresponding anti-BxACE polyclonal antibody (BxACEPab) from the *B. xylophilus* crude protein samples extracted in the presence (lane 1) or absence (lane 2) of Triton X-100.

The specificity of three BxACEPabs for immunohistochemistry was determined by Western blot using recombinant BxAChEs. Little cross-reactivity was observed among the three BxACEPabs (Fig. S1). BxACE-1 was observed in several histological regions of *B. xylophilus* (Fig. 1A). BxACE-1-specific fluorescent signals were detected in the nerve ring (NR) around the pharynx, where most sensory integration takes place, the putative ventral nerve cord (VNC), the preanal ganglia region (PGA) and the dorsal nerve cord (DNC). BxACE-2 was detected in limited regions that were different from those expressing BxACE-1 (Fig. 1B). BxACE-2 was mostly observed around the NR, particularly at the post-NR, which appears to be the retrovesicular ganglia (RVG), a cluster of 13 neuronal cell bodies in the VNC [21], and at the vulva of females, but not at the spiculus of males. BxACE-1 and BxACE-2 signals were mostly non-overlapping. BxACE-3 was observed around the putative hypodermal cells (HC) at the end of the head (Fig. 1C) and the tip of the tail, which cover the major opening of the body. In addition, BxACE-3 was observed at the putative intestinal regions and the canal-associated neuron (CAN), where BxACE-1 and BxACE-2 were not detected. BxACE-3 was not observed around the NR.

Native PAGE using the crude proteins extracted with buffer with or without Triton-X 100 followed by Western blot revealed that a large fraction of BxACE-3 was successfully extracted with the buffer without detergent. In contrast, relatively much reduced amounts of BxACE-1 or BxACE-2 were extracted without detergent. These findings suggest that a large part of BxACE-3 is not associated with a lipid anchor and exists mostly in the soluble fraction unlike the membrane-anchored BxACE-1 and BxACE-2, further suggesting the distribution of BxACE-3 in extracellular space (Fig. 1D).

In fact, BxACE-3 does not have a GPI-anchoring sequence or an H-type hydrophobic sequence [17], suggesting that it is likely secreted to the extracellular space or to the hemocoel. Presence of soluble form AChE3 has not been confirmed in other nematodes, including the free-living nematode *Caenorhabditis elegans* and some phytopathogenic nematodes such as *H. glycines*, *M. arenaria* and *M. incognita* assumed membrane-associated protein [7], [15], [16], [22], [23].

### Presence of BxACE-3 reduces the BxACE inhibition by anti-cholinesterase compounds

**Figure 2:**
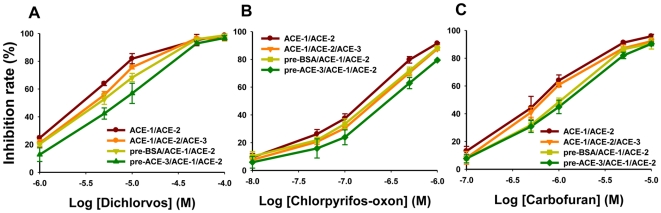
Inhibition rate of dichlorvos (A), chlorpyrifos-oxon (B) and carbofuran (C) in the presence or absence of BxACE-3. For all of the tested inhibitors, the enzyme mixture without BxACE-3 (brown line) was the most inhibited, whereas the enzyme mixture with BxACE-3 pre-incubated for 10 min with inhibitors (green line) was the least inhibited. The enzyme mixture mixed at the same time (orange line) and the enzyme mixture with BSA pre-incubated for 10 min with inhibitors (yellow line) showed middle inhibition. The inhibition rates in response to chlorpyrifos-oxon, dichlorvos and carbofuran were statistically different between enzyme mixtures without BxACE-3 and with BxACE-3, as determined by GLM repeated measures ANOVA (p<0.05).

Based on the assumption that a large fraction of BxACE-3 is present in a soluble form in the extracellular space or/and body fluid, we tested the hypothesis that BxACE-3 plays a role in chemical defense against xenobiotics as in the case of vertebrate BuChE [24]. The inhibition rate of three anti-cholinesterase inhibitors (dichlorvos, chlorpyrifos-oxon and carbofuran) was measured by *in vitro* assay using a pooled preparation of purified BxACE-1 and BxACE-2 with or without the addition of BxACE-3 (Fig. 2). When the mixture of BxACE-1 and BxACE-2 was inhibited with diagnostic doses of the inhibitors, the overall inhibition was always significantly reduced (1.9- to 2.3-fold based on IC_50_) in the presence of BxACE-3 as determined by GLM repeated measures ANOVA (p<0.05) (Table S2). In particular, the lowest inhibition rate was observed when inhibitors were pre-incubated with BxACE-3 for 10 min and then added to the mixture of BxACE-1 and BxACE-2. The addition of BSA to the mixture of BxACEs also reduced the rate of inhibition, though the rates of reduction were lower than those obtained by the addition of BxACE-3. Because BxACE-3 activity itself was negligible, the addition of BxACE-3 to the mixture of BxACE-1 and BxACE-2 did not likely contribute to the remaining AChE activity measured after inhibition. In fact, overall control AChE activity was not changed by the addition of BxACE-3 to the mixture of BxACE-1 and BxACE-2. Therefore, the significant reduction in AChE inhibition observed in the presence of BxACE-3 indicates that BxACE-3 protects BxACE-1 and BxACE-2 from inhibition, perhaps by sequestering inhibitors.

BxACE-3 exhibited the highest transcription level in all of the life stages [17], which is contrast to *C. elegans* in which the transcript level of orthologous AChE was similar to other AChEs as estimated by Northern blotting [7]. BxACE-3 also showed the lowest catalytic efficiency toward several substrates in spite of its high affinity to them [17], implying that it is not likely involved in the hydrolysis of neurotransmitter. These findings further suggest that BxACE-3 function as a ubiquitous sequestration protein, rather than as a specific hydrolase, by binding nonspecifically to a variety of xenobiotics. Taken together, BxACE-3 appears to play little role in postsynaptic transmission but rather has non-classical functions, such as protection of the nervous system from toxic xenobiotics, probably by sequestering them.

In this sense, BxACE-3 resembles the vertebrate butyrylcholinesterase (BuChE, EC 3.1.1.8), which serves as an effective sequestering agent against toxins or toxicants before they arrive at their target sites [25], [26]. The BuChE is known to have several functions, including a backup for AChE, an auxiliary role in development and a protective function in detoxifying orally ingested toxic compounds [27], [28], [29]. This soluble enzyme is observed at high concentrations in plasma, liver, lung and intestine [30] and expressed ten times more than AChE in humans [31]. It is generally agreed that BuChE removes the artificial or natural anti-cholinesterase inhibitors by being irreversibly inhibited by them [32], [33]. In fact, intravenous administration of human BuChE to figs or monkeys increases LD_50_ against nerve agents, demonstrating its protective function [25], [26], [34]. Although BuChE appears to be diverged from AChE in early chordate lineage [35] and to belong to a completely separate lineage from BxACE-3 [17], it is interesting that both vertebrate BuChE and nematode BxACE-3 share a similar physiological function as a bioscavenger providing chemical defense against xenobiotics.

### Bxace-3 knockdown increases the *B. xylophilus* sensitivity to three nematicidal compounds

**Figure 3:**
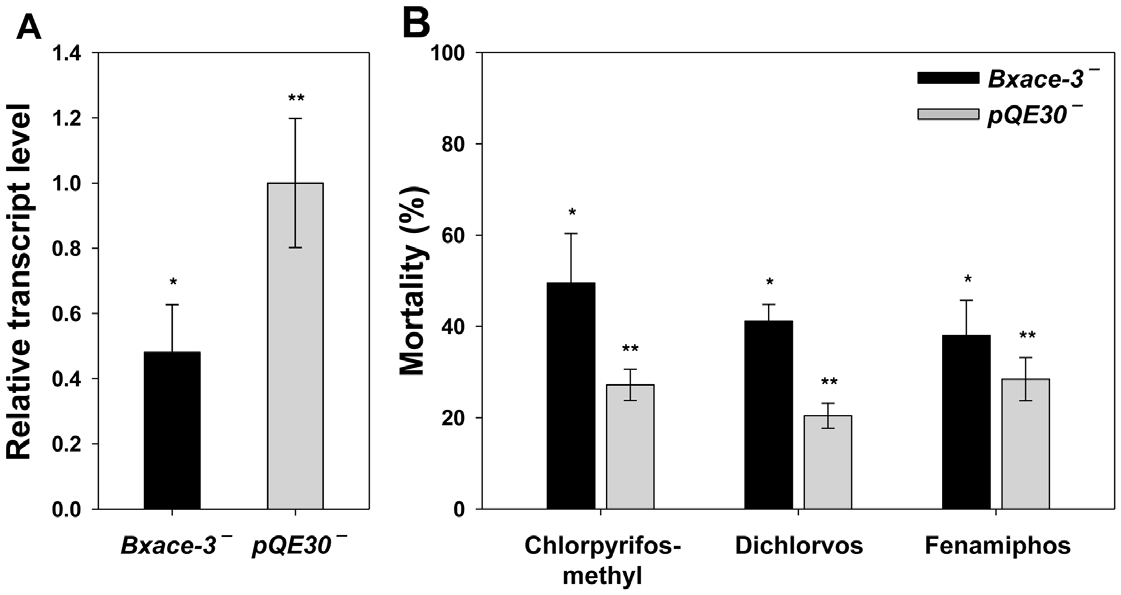
The relative transcript level of *Bxace-3* determined by qPCR (A) and mortality in response to chlorpyrifos-methyl, dichlorvos and fenamiphos (B) in *pQE30^**–**^* and *Bxace-3^**–**^* nematodes. The relative transcript level of *Bxace-3* and the mortality in response to chlorpyrifos-methyl, dichlorvos and fenamiphos were significantly different between *pQE30^–^*- and *Bxace-3^–^* nematodes, as determined by *t*-test (p<0.05).

BxACE-3 transcription in the nematodes treated with *Bxace-3* dsRNA (*Bxace-3^–^*) was 48% suppressed compared to the nematodes treated with pQE30 dsRNA (*pQE30* ^–^) (Fig. 3A). Nevertheless, no abnormal phenotype or mobility was observed in the *Bxace-3^–^* nematodes (data not shown). In bioassays using chlorpyrifos-methyl, dichlorvos and fenamiphos, however, the mortality of *Bxace-3^–^* nematodes (49.5, 41.2 and 38.0%) was 1.3–2.0-fold higher than that of *pQE30* ^–^ nematodes (27.2, 20.4 and 28.4%), demonstrating that knowndown of *Bxace-3* increased the mortality of *B. xylophilus* (Fig. 3B). These lines of evidence confirm the role of BxACE-3 in chemical defense against various xenobiotics, including nematicides.

### BxACE-3 interacts with endogenous pine resin terpenes

Constant exposure of the monophagous *B. xylophilus* to the toxic secondary compounds of pine tree hosts may have driven BxACE-3 to adapt to such a chemical defense function. *B. xylophilus* spends most of its life inside pine tree except for migration via insect vectors and must counteract against various physical and chemical defenses by the host pine tree, particularly in the propagative stage. Pine resin is a major defense compound against various pests, including insects, nematodes and fungi [36], [37] and is composed of several terpenes, toxic primary metabolites. The α-pinene (bicyclic terpene) and limonene (monocyclic terpene), which are the most common constituents of pine resin, are known to be AChE inhibitors [38], [39], [40], [41], [42]. Therefore, it can be postulated that *B. xylophilus* has evolved the chemical defense system based on BxACE-3 against the anti-AChE terpene compounds.

**Figure 4:**
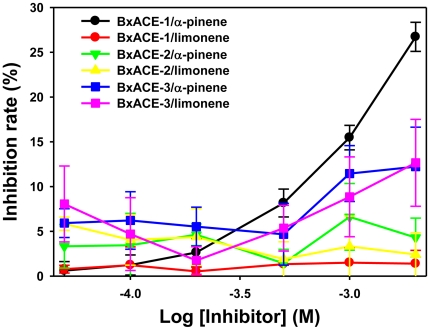
Inhibition test using α-pinene and limonene. BxACE-1 was over 25% inhibited by α-pinene (black line and filled circle) but not by limonene (red line and filled circle), and BxACE-3 was somewhat inhibited by the two inhibitors (α-pinene: blue line and filled square; limonene: violet line and filled square). BxACE-2 was not inhibited (α-pinene: green line and filled inverted triangle; limonene: yellow line and filled triangle).

To test this hypothesis, AChE inhibition assays using α-pinene and limonene were conducted. α-pinene inhibited BxACE-1 in a dose-dependent manner (Fig. 4). BxACE-1 activity was inhibited approximately 25% by 2 mM α-pinene but not by limonene. BxACE-2 was not inhibited by these compounds but BxACE-3 activity was inhibited ∼10% by 2 mM α-pinene and limonene. Inhibition of BxACE-3 by both α-pinene and limonene indicates that BxACE-3 interacts with these secondary toxins, thereby protecting the main postsynaptic BxACE-1 from inhibition. The higher level of expression in the pine-grown stage and the specific distribution around the end of head, the tip of tail and the canal, the first barriers against xenobiotics, further imply a chemical defense function of BxACE-3 against the secondary pine tree toxins. Considering that the free-living *C. elegans* ACE-3 shows different expression patterns from BxACE-3 [9], the chemical defense function of BxACE-3 is likely unique to phytophagous nematodes such as *B. xylophilus*.

### Functional diversification of BxACE-3 as a bioscavenger

AChE is presumed to be generated before cnidarians, but ancient AChE appears to have non-neuronal functions because AChE of cnidarians shows a low catalytic activity and is mostly expressed in non-nervous tissues [43], [44]. It is interesting that AChE in cnidarians is mainly associated with non-neuronal functions although they have the postsynaptic nervous system [45], [46]. These facts suggest that early AChEs did not play roles related to neuronal function and the association of AChE to postsynaptic transmission was an event after the evolution of cnidarian. In fact, the generation of proteins having the activity in nervous tissues and the structural features of the active classical AChE appears in the ancient bilaterian, such as some flatworms and nematodes [47], [48]. Based on this, it is highly probable that the Nematoda is an intermediate phylum in the early bilaterian having both neuronal and non-neuronal AChEs. In case of *B. xylophilus*, both BxACE-1 and BxACE-2, which have relatively high catalytic activities, are involved in the postsynaptic transmission, whereas BxACE-3 appears to adapt to a non-neuronal role of chemical defense against xenobiotics. The role of BxACE-3 as a bioscavenger provides important insights into the functional diversification of AChE.

## Supporting Information

Figure S1 **The cross activity assay of three BxACEPabs against recombinant BxACEs expressed by baculovirus system through Western Blot**. Each BxACEPab shows no cross activity against other BxACEs.
(PDF)

Table S1 **The list of primers for synthesis of dsRNA used in RNAi.**
(PDF)

Table S2 **The IC_50_ of several BxACE mixtures against three nematicidal reagents.**
(PDF)

## References

[1] Massoulie J, Pezzementi L, Bon S, Krejci E, Vallette FM (1993). Molecular and Cellular Biology of Cholinesterases.. Progress in Neurobiology.

[2] Balasubramanian AS, Bhanumathy CD (1993). Noncholinergic functions of cholinesterases.. FASEB Journal.

[3] Martelly I, Gautron J (1988). Differential expression of acetylcholinesterase molecular forms in neural retina and retinal pigmented epithelium during chick development.. Brain Research.

[4] Thullbery MD, Cox HD, Schule T, Thompson CM, George KM (2005). Differential localization of acetylcholinesterase in neuronal and non-neuronal cells.. Journal of Cellular Biochemistry.

[5] Alles GA, Hawes RC (1940). Cholinestebases in the blood of man.. Journal of Biological Chemistry.

[6] Augustinsson KB (1948). On the specificity of cholinesterase.. Biology Bulletin.

[7] Combes D, Fedon Y, Grauso M, Toutant JP, Arpagaus M (2000). Four genes encode acetylcholinesterases in the nematodes *Caenorhabditis elegans* and *Caenorhabditis briggsae*. cDNA sequences, Genomic structures, mutations and in vivo expression.. Journal of Molecular Biology.

[8] Toutant JP (1989). Insect acetylcholinesterase: catalytic properties, tissue distribution and molecular forms.. Progress in Neurobiology.

[9] Combes D, Fedon Y, Toutant JP, Arpagaus M (2003). Multiple ace genes encoding acetylcholinesterases of *Caenorhabditis elegans* have distinct tissue expression.. European Journal of Neuroscience.

[10] Culetto E, Combes D, Fedon Y, Roig A, Toutant JP (1999). Structure and promoter activity of the 5′ flanking region of *ace-1*, the gene encoding acetylcholinesterase of class a in *Caenorhabditis elegans*.. Journal of Molecular Biology.

[11] Culotti JG, Vonehrenstein G, Culotti MR, Russell RL (1981). A 2nd class of acetylcholinesterase-Deficient mutants of the nematode *Caenorhabditis elegans*.. Genetics.

[12] Johnson CD, Duckett JG, Culotti JG, Herman RK, Meneely PM (1981). An acetylcholinesterase deficient mutant of the nematode *Caenorhabditis elegans*.. Genetics.

[13] Combes D, Fedon Y, Toutant JP, Arpagaus M (2001). Acetylcholinesterase genes in the nematode *Caenorhabditis elegans*.. International Review of Cytology.

[14] Selkirk ME, Lazari O, Matthews JB (2005). Functional genomics of nematode acetylcholinesterases.. Parasitology.

[15] Chang S, Opperman CH (1991). Characterization of acetylcholinesterase molecular forms of the root knot nematode, *Meloidogyne*.. Molecular and Biochemical Parasitology.

[16] Chang S, Opperman CH (1992). Separation and characterization of *Heterodera glycines* acetylcholinesterase molecular forms.. Journal of Nematology.

[17] Kang JS, Lee DW, Choi JY, Je YH, Koh YH (2011). Three acetylcholinesterases of the pinewood nematode, *Bursaphelenchus xylophilus*: Insights into distinct physiological functions.. Molecular & Biochemical Parasitology.

[18] Kang JS, Moon IS, Lee SG, Shin SC, Lee SH (2009). Rapid and accurate prediction of the frequencies of *Bursaphelenchus xylophilus* and *B. mucronatus* in mixed nematode samples using real-time species-specific PCR.. Nematology.

[19] Wood WB, Herman RK, Emmons SW, White J, Sulston J (1988). The Nematode *Caenorhabditis elegans*..

[20] Duerr JS, Community TCeR (2006). Immunohistochemistry.. Wormbook: Wormbook.

[21] Angstadt JD, Donmoyer JE, Stretton AOW (1989). Retrovesicular ganglion of the nematode *Ascaris*.. Journal of Comparative Neurology.

[22] Kolson DL, Russell RL (1985). A novel class of acetylcholinesterase, revealed by mutations, in the nematode *Caenorhabditis elegans*.. Journal of Neurogenetics.

[23] Kolson DL, Russell RL (1985). New acetylcholinesterase-deficient mutants of the nematode *Caenorhabditis elegans*.. Journal of Neurogenetics.

[24] Lockridge O, Masson P (2000). Pesticides and susceptible populations: People with butyrylcholinesterase genetic variants may be at risk.. Neurotoxicology.

[25] Lenz DE, Maxwell DM, Koplovitz I, Clark CR, Capacio BR (2005). Protection against soman or VX poisoning by human butyrylcholinesterase in guinea pigs and cynomolgus monkeys.. Chemico-Biological Interactions.

[26] Raveh L, Grauer E, Grunwald J, Cohen E, Ashani Y (1997). The stoichiometry of protection against soman and VX toxicity in monkeys pretreated with human butyrylcholinesterase.. Toxicology and Applied Pharmacology.

[27] Ladu BN, Bartels CF, Nogueira CP, Arpagaus M, Lockridge O (1991). Proposed nomenclature for human butyrylcholinesterase genetic variants identified by DNA sequencing.. Cellular and Molecular Neurobiology.

[28] Layer PG (1991). Cholinesterases during development of the avian nervous system.. Cellular and Molecular Neurobiology.

[29] Masson P, Lockridge O (2010). Butyrylcholinesterase for protection from organophosphorus poisons: catalytic complexities and hysteretic behavior.. Archives of Biochemistry and Biophysics.

[30] Lazari O, Hussein AS, Selkirk ME, Davidson AJ, Thompson FJ (2003). Cloning and expression of two secretory acetylcholinesterases from the bovine lungworm, *Dictyocaulus viviparus*.. Molecular and Biochemical Parasitology.

[31] Manoharan I, Boopathy R, Darvesh S, Lockridge O (2007). A medical health report on individuals with silent butyrylcholinesterase in the Vysya community of India.. Clinica Chimica Acta.

[32] Casida JE, Quistad GB (2004). Organophosphate toxicology: safety aspects of nonacetylcholinesterase secondary targets.. Chemical Research in Toxicology.

[33] Duysen EG, Li B, Darvesh S, Lockridge O (2007). Sensitivity of butyrylcholinesterase knockout mice to (--)-huperzine A and donepezil suggests humans with butyrylcholinesterase deficiency may not tolerate these Alzheimer's disease drugs and indicates butyrylcholinesterase function in neurotransmission.. Toxicology.

[34] Saxena A, Sun W, Luo C, Myers TM, Koplovitz I (2006). Bioscavenger for protection from toxicity of organophosphorus compounds.. Journal of Molecular Neuroscience.

[35] Pezzementi L, Chatonnet A (2010). Evolution of cholinesterases in the animal kingdom.. Chemico-Biological Interactions in press.

[36] Phillips MA, Croteau RB (1999). Resin-based defenses in conifers.. Trends in Plant Science.

[37] Trapp S, Croteau R (2001). Defensive Resin Biosynthesis in Conifers.. Annual Review of Plant Physiology and Plant Molecular Biology.

[38] Kumar P, Singh VK, Singh DK (2009). Kinetics of enzyme inhibition by active molluscicidal agents ferulic acid, umbelliferone, eugenol and limonene in the nervous tissue of snail *Lymnaea acuminata*.. Phytotherapy Research.

[39] Lim JH, Kim JC, Kim KJ, Son YS, Sunwoo Y (2008). Seasonal variations of monoterpene emissions from Pinus densiflora in East Asia.. Chemosphere.

[40] Miyazawa M, Watanabe H, Kameoka H (1997). Inhibition of acetylcholinesterase activity by monoterpenoids with a p-menthane skeleton.. Journal of Agricultural and Food Chemistry.

[41] Miyazawa M, Yamafuji C (2005). Inhibition of acetylcholinesterase activity by bicyclic monoterpenoids.. Journal of Agricultural and Food Chemistry.

[42] Perry NSL, Houghton PJ, Theobald A, Jenner P, Perry EK (2000). In vitro inhibition of human erythrocyte acetylcholinesterase by *Salvia lavandulaefolia* essential oil and constituent terpenes.. Journal of Pharmacy and Pharmacology.

[43] Denker E, Chatonnet A, Rabet N (2008). Acetylcholinesterase activity in Clytia hemisphaerica (Cnidaria).. Chemico-Biological Interactions.

[44] Takahashi T, Hamaue N (2010). Molecular characterization of Hydra acetylcholinesterase and its catalytic activity.. FEBS Letters.

[45] Nickel M (2010). Evolutionary emergence of synaptic nervous systems: what can we learn from the non-synaptic, nerveless Porifera?. Invertebrate Biology.

[46] Ryan TJ, Grant SG (2009). The origin and evolution of synapses.. Nature Reviews Neuroscience.

[47] Bentley GN, Jones AK, Agnew A (2005). Expression and comparative functional characterisation of recombinant acetylcholinesterase from three species of Schistosoma.. Molecular & Biochemical Parasitology.

[48] Bery A, Cardona A, Martinez P, Hartenstein V (2010). Structure of the central nervous system of a juvenile acoel, *Symsagittifera roscoffensis*.. Development Genes and Evolution.

