# Supplementary figures and images for: A Soluble Acetylcholinesterase Provides Chemical Defense against Xenobiotics in the Pinewood Nematode
Source: PLoS One. 2011 Apr 27;6(4):e19063. doi: 10.1371/journal.pone.0019063 (PMC3083410; doi:10.1371/journal.pone.0019063)

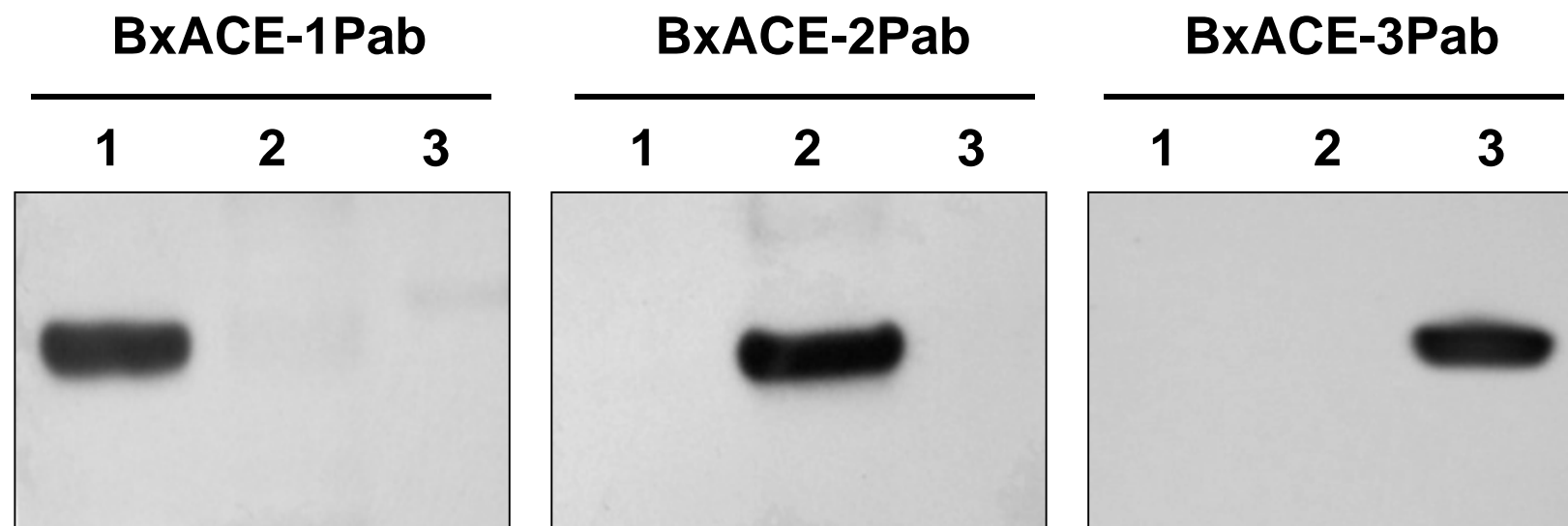

Supplement: Figure S1 — The cross activity assay of three BxACEPabs against recombinant BxACEs expressed by baculovirus system through Western Blot. Each BxACEPab shows no cross activity against other BxACEs. (PDF) [file pone.0019063.s001.pdf]
