# Supplementary material for: A Soluble Acetylcholinesterase Provides Chemical Defense against Xenobiotics in the Pinewood Nematode
Source: PLoS One. 2011 Apr 27;6(4):e19063. doi: 10.1371/journal.pone.0019063 (PMC3083410; doi:10.1371/journal.pone.0019063)
Supplement: Table S1 — The list of primers for synthesis of dsRNA used in RNAi. (PDF) [file pone.0019063.s002.pdf]

Genes	Name	Sequences[a]
Bxace-3	*5Bxace-3*siRNA	**TAATACGACTCACTATAGGGAGA**GCTACCAGCAGAGTCTGCCCATAAT
	3*Bxace-3*siRNA	**TAATACGACTCACTATAGGGAGA**TTTGAGGCAGTCCAATATGTACTG
pQE30	5*pQE30*siRNA	**TAATACGACTCACTATAGGGAGA**CCCCGTTTTCACCATGGGCAAATA
	3*pQE30*siRNA	**TAATACGACTCACTATAGGGAGA**ATTCTGCCGACATGGAAGCCATCA

[a] Bold sequences indicate T7 promoter sequence for synthesis of dsRNA.
