# Supplementary material for: A Soluble Acetylcholinesterase Provides Chemical Defense against Xenobiotics in the Pinewood Nematode
Source: PLoS One. 2011 Apr 27;6(4):e19063. doi: 10.1371/journal.pone.0019063 (PMC3083410; doi:10.1371/journal.pone.0019063)
Supplement: Table S2 — The IC50 of several BxACE mixtures against three nematicidal reagents. (PDF) [file pone.0019063.s003.pdf]

	Enzyme mixtures	Chlorpyrifos-oxon (10^{-6} M)	Dichlorvos (10^{-5} M)	Carbofuran (10^{-5} M)
IC_{50}	ACE-1+ACE-2	0.12	0.28	0.06
	ACE-1+ACE-2+ACE-3	0.17	0.36	0.08
	ACE-1+ACE-2+pre[a]-BSA	0.15	0.42	0.10
	ACE-1+ACE-2+pre-ACE-3	0.25	0.65	0.12
IC_{50} ratio	(ACE-1+ACE-2+ACE-3)/(ACE-1+ACE-2)	1.4	1.3	1.3
	(ACE-1+ACE-2+pre-ACE-3)/(ACE-1+ACE-2)	2.0	2.3	1.9

[a] Indicates pre-incubation for 10 min before adding to mixture of ACE-1 and ACE-2.
